# CHIKV-Infected Human Dermal Fibroblasts Mount an IFNβ Transcriptional Response Independent of TBK1/IKKε Signaling That Fails to Prevent Lethal Infection

**DOI:** 10.3390/v18050503

**Published:** 2026-04-28

**Authors:** Meagan M. Taylor, Rosemary W. Roberts, Jonathan O. Rayner

**Affiliations:** Department of Microbiology & Immunology, Frederick P. Whiddon College of Medicine, University of South Alabama, Mobile, AL 36688, USA; mmt1322@jagmail.southalabama.edu (M.M.T.);

**Keywords:** CHIKV, Aedes, dermal fibroblasts, TBK1/IKKε, IFNβ, alphavirus, arbovirus

## Abstract

Chikungunya virus (CHIKV) is an alphavirus that infects dermal fibroblasts as a primary target cell during natural mosquito-borne transmission. While primary human dermal fibroblasts (hDFs) have been implicated as a key source of type I interferon (IFN-I) during CHIKV infection, the dynamics of this response and its sufficiency for antiviral protection remain incompletely understood. Here, we systematically characterize in vitro CHIKV infection of primary hDFs, evaluating the effects of single-passage viral stock origin (mammalian- vs. mosquito-propagated), donor variability, and multiplicity of infection (MOI) on infection kinetics and innate immune induction. We demonstrate that hDFs support high-titered CHIKV replication at both MOI 1 and 0.01, resulting in universal cell death by 72 hpi despite robust IFNβ transcript induction—reaching up to ~2800-fold over mock—and secretion of pro-inflammatory cytokines, including IFNα2, TNFα, IL-1β, and IL-8. Notably, IFNβ protein levels remained below 10 pg/mL under all infection conditions, revealing a disconnect between transcriptional and translational responses, suggesting CHIKV-mediated translational suppression. Pharmacological inhibition of TBK1/IKKε via amlexanox did not suppress IFNβ transcript induction at any tested concentration, suggesting that canonical PRR signaling through this node—including both RIG-I/MAVS and TLR3/TRIF pathways—is not the major driver of the observed transcriptional response. In contrast, co-inoculation with exogenous IFNβ as low as 20 pg/mL activated IFNAR signaling, robustly upregulated interferon-stimulated genes (ISGs), and fully rescued hDFs from otherwise lethal infection. Together, these findings demonstrate that CHIKV-infected hDFs mount a transcriptionally robust but translationally insufficient innate immune response and that the transcriptional response appears to operate independently of TBK1/IKKε. These results have direct implications for understanding how the skin microenvironment may modulate early CHIKV pathogenesis and suggest that paracrine IFNβ signaling from neighboring cell types may be critical for fibroblast survival during natural infection.

## 1. Introduction

Chikungunya virus (CHIKV) is a mosquito-borne alphavirus in the *Togaviridae* family. Initially discovered in modern day Tanzania in 1952, CHIKV has since spread globally and represents a significant public health threat [1]. In fact, over 8000 confirmed CHIKV cases were reported in Foshan, China from June to August 2025, highlighting the explosive nature of CHIKV outbreaks [1,2,3]. Currently, two vaccines have received Food and Drug Administration (FDA) approval in the US to prevent CHIKV infection; however, one of these vaccines—IXCHIQ, a live attenuated CHIKV vaccine—had its approval suspended in August 2025 due to reports of severe CHIKV-like symptoms [3,4]. The other—VIMKUNYA, a virus-like particle vaccine—is still being studied for efficacy but retains its FDA approval [3,5]. Furthermore, there are no approved therapeutics to treat CHIKV infection once symptoms arise, leaving infected patients with limited options for symptom relief. CHIKV infection is predominantly marked by febrile symptoms, such as fever (~88%) and headache (~50%), but the hallmark indicator is debilitating arthralgia (~90%) [6,7]. “Chikungunya” translates to “that which bends” in the Kimakonde dialect and originates from the hunched posture exhibited by CHIKV-infected individuals [1]. Up to 50% of patients will continue experiencing severe arthralgia for months to years beyond initial infection, with women, those of advanced age, and immunocompromised individuals being most at risk [6,7,8,9,10]. Severe CHIKV cases are characterized by multi-organ failure and death. Though rarely fatal, CHIKV deaths are increasing as the virus reaches previously vulnerable populations and reportedly more prevalent in individuals of the male sex [11,12,13].

During early CHIKV infection of immunocompetent mice via needle inoculation, a robust type I interferon response is activated, resulting in decreased viral dissemination and prevention of human-like pathology. IFN-I signaling triggers upregulation of interferon-stimulated gene (ISG) expression like ISG15, interferon-inducible protein 6 (IFI6), interferon-induced transmembrane proteins (IFITMs), and 2′-5′-oligoadenylate synthetase-like protein (OASL) in human cell lines [14,15]. CHIKV also causes production and secretion of proinflammatory cytokines into the sera of human patients, including interleukin 1 beta (IL-1β), interleukin 6 (IL-6), and interleukin 8 (IL-8), which recruit immune cells to the site [16]. Whether primary human skin cells mount a comparably effective innate immune response, however, remains incompletely understood. Most notably, previous studies implicate human dermal fibroblasts as a primary source of the innate immune response in CHIKV-infected humans [17,18,19,20,21].

Despite this transcriptional response, it remains unclear why hDFs succumb to CHIKV infection rather than establishing an effective antiviral state. One possibility is that CHIKV-mediated host shutoff prevents sufficient translation of IFNβ protein, limiting autocrine IFNAR signaling below the threshold required for protection. Additionally, the pattern recognition receptor (PRR) primarily responsible for driving *IFNβ* transcription in primary hDFs specifically has not been directly interrogated. RIG-I and TLR3 are both implicated in sensing CHIKV RNA in human fibroblasts, with both pathways converging on TBK1/IKKε to drive *IFNβ* transcription [17,20,21,22,23,24,25,26]. Understanding whether canonical PRR signaling through this node drives the robust transcriptional response observed in hDFs—and whether these cells retain the capacity to respond to IFNβ even if they are unable to produce it effectively—is critical for interpreting early infection dynamics in human skin.

Notably, mosquito saliva has been shown to modulate the skin immune environment during arboviral transmission. Studies comparing needle- versus mosquito-delivered CHIKV have demonstrated that salivary components can suppress IFN-I signaling in dermal fibroblasts and alter immune cell recruitment at the inoculation site [27,28,29]. While the present study does not directly examine salivary components, the infection system characterized here provides a necessary foundation for future investigation of how mosquito saliva may modulate the early innate immune response to CHIKV in human skin.

In this study, we systematically characterize CHIKV infection of primary hDFs under conditions relevant to natural transmission. Specifically, we compared infection kinetics using viral stocks propagated in either mammalian or mosquito cell lines; assessed reproducibility across hDF donors; and evaluated the impact of MOI on viral replication and innate immune timing. To interrogate the PRR pathway driving *IFNβ* transcription, we treated CHIKV-infected hDFs with amlexanox, a pharmacological inhibitor of TBK1 and IKKε, which serve as convergent downstream effectors of both RIG-I/MAVS and TLR3/TRIF signaling. Finally, to determine whether hDFs retain functional IFNβ signaling capacity despite failing to survive infection, we co-inoculated cells with exogenous IFNβ and assessed antiviral gene expression and viral suppression. Together, these studies reveal a critical disconnect between *IFNβ* transcriptional induction and translational output during CHIKV infection and provide evidence that this transcriptional response operates independently of TBK1/IKKε.

## 2. Materials and Methods

### 2.1. Cells

Two different cell lines of primary human dermal fibroblasts (hDFs) were purchased from ATCC (CCD-1123Sk and CCD-1135Sk, see Supplementary Materials File S1 for details). These cells were obtained from different adult patients’ healthy skin. Complete media consisted of Dulbecco’s Modified Eagle Medium (DMEM) (Corning, Corning, NY, USA; 15-013-CV) with 10% FBS, 1% penicillin/streptomycin (Gibco, Waltham, MA, USA; 15180-122), and 1% L-glutamine (Gibco, 25030-081). hDFs were maintained at 37 °C and 5% CO_2_ in filter-cap tissue culture flasks. Vero E6 cells (African green monkey kidney cells) were obtained from ATCC (CRL-1586, RRID: CVCL_0574) and grown under the same conditions as hDFs. C6/36 cells were cultured in L15 media (Gibco, 11415-064) with 10% heat-inactivated FBS and 1% penicillin/streptomycin at 28 °C in plug-cap flasks.

### 2.2. Viruses

CHIKV LR2006-OPY1 was kindly gifted by Drs. Steven Higgs and Dana Vanlandingham at Kansas State University. CHIKV strains were expanded under a single passage in either Vero E6 cells (referred to here as mammalian-propagated stocks) or C6/36 cells (referred to here as mosquito-propagated stocks) using an MOI of 0.01. Then, supernatants were harvested at 48 hpi by collecting entire contents of supernatant, filtering through a 0.45 μm syringe filter, and then aliquoting cryovials for storage at −80 °C. Stock titers were obtained using TCID_50_, as well as RT-qPCR of supernatants. Master or working viral stocks were analyzed for endotoxin and mycoplasma contamination prior to use in infection protocols.

### 2.3. Compounds and Proteins

Recombinant human IFNβ was obtained from Fisher Scientific (PeproTech, Cranbury, NJ, USA; 300-02BC-5UG) and received lyophilized. Aliquots were prepared following reconstitution in DMEM with 0.1% bovine serum albumin (BSA) to 250 ng/mL and stored at −20 °C until immediately prior to inoculum and maintenance media preparation. Amlexanox, a selective TBK1/IKKε inhibitor, was obtained from BioTechne (Tocris, Bristol, UK; 4857) and also received in lyophilized form. Aliquots were prepared following reconstitution in tissue-culture-grade DMSO (freshly opened) to 10 mM. Both amlexanox and recombinant IFNβ aliquots were stored at −20 °C until immediately prior to inoculum and maintenance media preparation.

### 2.4. hDF Infection with CHIKV

hDFs were seeded in 12-well tissue-culture-treated plates (Greiner Bio-One, Kremsmünster, Austria; 665180) at approximately 50,000 to 60,000 cells per mL with 1 mL per well. Upon reaching approximately 70% confluency, at least three wells of a 12-well plate were counted and used to calculate cells per well for accurate multiplicity of infection (MOI) calculations. MOIs of either 1 or 0.01 were used in this study and are noted on each figure or in figure captions. At the indicated timepoints, cells were observed for cytopathic effects (CPE) (phenotypically observed as cell rounding and detachment from culture surface—representative images in Appendix A), and supernatants were collected for CHIKV infectious titer determination. Additionally, monolayers were collected by lysing with RNAzol RT (Molecular Research Center, Cincinnati, OH, USA; RN190) in approximately 0.1 mL of RNAzol per cm^2^ surface area. Supernatants were stored at −80 °C, and cell lysates were stored at −20 °C. For infections where cells were co-inoculated with recombinant human IFNβ (PeproTech, 300-02BC-5UG) or amlexanox (Tocris, 4857), the same methods were used as above. Maintenance media with either BSA or DMSO were prepared to serve as vehicle controls for IFNβ or amlexanox, respectively. Appropriate volumes of viral stocks were diluted in maintenance media to achieve the desired MOI.

### 2.5. Infectious Titers by 50% Tissue Culture Infectious Dose

Infectious titers were determined by 50% Tissue Culture Infectious Dose (TCID_50_). Vero E6 cells were plated at 2 × 10^5^ cells/mL in 0.1 mL per well and grown overnight in 96-well tissue-culture-treated microplates (Alkali Scientific CellPro, Ft. Lauderdale, FL, USA; TPN1096) at 37 °C with 5% CO_2_ in complete media (DMEM + 10% FBS). A separate dilution plate (CellTreat, Ayer, MA, USA; 229597) was prepared to serially dilute supernatants by adding 180 μL of maintenance media (DMEM + 2% FBS) to each well. Then, 20 μL of supernatant was added to at least three consecutive wells and then serially diluted 10-fold down the plate. Cell culture plate media were removed and then replaced with 100 μL of diluted supernatant to corresponding wells with a multichannel pipette. Cells were then incubated at 37 °C with 5% CO_2_ for at least 120 h before scoring for presence or absence of CPE. TCID_50_ titers were calculated using the Reed–Muench method (Supplementary Materials File S3).

### 2.6. RNA Extraction from Adherent Monolayers

Monolayers from all wells were lysed with RNAzol RT (Molecular Research Center, Cincinnati, OH, USA; RN190) at 0.1 mL per cm^2^ well surface area (350 μL per well) at the indicated timepoints. To ensure complete lysis, RNAzol RT was added to each well and then incubated at room temperature for approximately 5 min. Then, lysates were collected by tilting the plate and using the RNAzol in each well to wash the monolayer. Then, to homogenize, lysates were pipetted up and down 10 times and moved to a microcentrifuge tube for storage at −20 °C until RNA extractions were performed. RNA extraction of the monolayers was performed using the Zymo Direct-zol MiniPrep Kit (Zymo Research, Irvine, CA, USA; R2052) according to the manufacturer’s instructions. RNA was eluted from spin columns in 60 µL of nuclease-free water and then stored at −80 °C until ready for use. RNA concentrations were determined through UV-Vis on a NanoDrop (ThermoFisher Scientific, Waltham, MA, USA; ND-1000) and then diluted in nuclease-free water to ~15 ng/μL to ensure each reaction had the same concentrations immediately prior to inclusion in one-step RT-qPCR or stored at −20 °C for up to 1 week.

### 2.7. RT-qPCR

RT-qPCR was performed using the New England BioLabs Luna Universal Probe One-Step RT-qPCR Kit (New England Biolabs, Ipswich, MA, USA; E3006) according to the manufacturer’s instructions. Primers/probes for human *IFNβ*, *TLR3*, *DDX58*, *IFIH1*, *ISG15*, and *GAPDH* were purchased from Integrated DNA Technologies (IDT; Coralville, IA, USA) (Supplementary Materials File S1) and supplied with a 2:1 ratio of primers to probes. Primers were reconstituted using IDTE pH 8.0 (IDT, Coralville, IA, USA; 11-01-03-01) at 40×, and then working stocks were diluted in a separate microfuge to 8×. All PCR reactions were performed in MicroAmp EnduraPlate Optical 96-well reaction plates (Applied Biosystems; Waltham, MA, USA). All RT-qPCR data were collected using the QuantStudio5 instrument (Applied Biosystems; Waltham, MA, USA) with Design and Analysis v1.5.3. All PCR reactions were prepared in a PCR workstation following UV treatment and RNase surface decontamination. Transcript expression as fold change was determined using the 2^−ΔΔCq^ method. Ct values were analyzed using QuantStudio Design and Analysis v2.8.0 software following RT-qPCR. The Excel template used for all 2^−ΔΔCq^ calculations is provided in the Supplementary Materials (Supplementary Materials File S2).

### 2.8. Cytokine Analysis by LEGENDplex

Cytokine analysis was performed using the LEGENDplex Human Anti-Virus Panel V02 according to the manufacturer’s instructions, including optional wash steps (BioLegend, San Diego, CA, USA; 741270) in a PlateOne 96-well V-bottom polystyrene plate (USA Scientific, Ocala, FL, USA; 1833-9600). Prior to removal from the BSL-3 laboratory, prepared LEGENDplex assays were treated with 4% PFA at room temperature for 10 min and then washed with 200 μL 1× wash buffer prior to resuspension. Data collection was performed using Agilent Novocyte Quanteon with Novocyte Q Autosampler (Agilent Technologies, Santa Clara, CA, USA). FCS files were then analyzed using the LEGENDplex data analysis suite online (BioLegend, v2025-05-01; https://legendplex.qognit.com/user/login?next=home; accessed on 1 February 2026). Concentrations were extrapolated using LEGENDplex online software (v2025-05-01) suite using plate-specific standard curves. Analytes with fewer than 100 beads were excluded from analysis.

### 2.9. Statistical Analysis

All data visualization and statistical analyses were performed with GraphPad Prism (v10.6.1, Macintosh). Data from two or more independent experiments were presented as mean ± standard error of the mean (SEM). Data from a single independent experiment were presented as mean ± standard deviation (SD). All viral titer data were first log-transformed and then statistical significance was assessed through 2-way ANOVA with Šidàk’s multiple comparison test. IFNβ fold-change values were analyzed on a linear scale, and then statistical significance was assessed through 2-way ANOVA with Šidàk’s multiple comparison test. Fold changes for IFNβ were determined by entering Ct values into the attached template for calculation of 2^−ΔΔCq^ (see Supplementary Materials File S2 for copy of Excel file used). TCID_50_ and RT-qPCR were completed for all samples and controls in triplicate. Samples and standards used in LEGENDplex assays were run in duplicate.

## 3. Results

### 3.1. Standardizing CHIKV Infection of hDFs

To establish a reproducible and biologically relevant primary hDF infection model for CHIKV, we systematically evaluated three variables known to influence in vitro infection dynamics: viral stock origin, hDF donor variability, and multiplicity of infection. The following subsections describe each comparison and the model decisions they informed. For all experiments described in this section, infectious titers were determined using TCID_50_, and *IFNβ* transcript expression was measured through RT-qPCR with *GAPDH* as the endogenous control, as previously validated for CHIKV-infected cells [30].

#### 3.1.1. Viral Stock Origin Influences Early CHIKV Infection Kinetics and IFNβ Transcript Induction in hDFs

**Figure 1:**
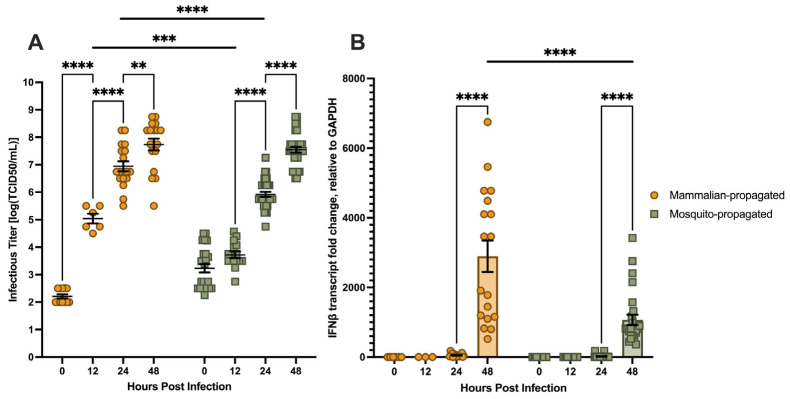
(**A**) Infectious CHIKV titers from the supernatants of infected hDFs at MOI 0.01 with stocks generated in Vero E6 (mammalian-propagated) or C6/36 (mosquito-propagated) determined through TCID_50_. (**B**) Relative fold change of *IFNβ* transcript expression (*GAPDH* as endogenous control) of CHIKV-infected hDFs measured through RT-qPCR. Bars represent the mean, and error bars represent SEM. Data symbols represent individual samples from at least three separate experiments (*n* = 3 to 33). The conditions with only 3 individual replicates were mammalian-derived IFNβ transcript expression at 0 and 12 hpi. Statistical analysis through 2-way ANOVA with Šidàk’s multiple comparison test. ** *p* < 0.01 *** *p* < 0.001 **** *p* < 0.0001.

To determine whether the cell source used to propagate viral stocks influences infection kinetics in hDFs, we compared stocks expanded in Vero E6 cells (mammalian-propagated) or C6/36 cells (mosquito-propagated) at MOI 0.01 over 48 hpi (Figure 1). Upon observation, CPE in CHIKV-infected hDFs was characterized by progressive cell rounding and detachment from the culture surface, with nearly all cells detaching from the culture vessel by 72 hpi (representative images in Appendix A.1).

Infectious titer accumulated more rapidly in hDFs infected with mammalian-propagated stocks than mosquito-propagated stocks (Figure 1A). Mammalian-propagated infections exhibited significant titer increases at each successive timepoint, from approximately 1.9 × 10^2^ TCID_50_/mL at 0 hpi to approximately 1.6 × 10^8^ TCID_50_/mL by 48 hpi. Mosquito-propagated infections also produced significant titer increases, but accumulation was delayed. No significant increase was observed at 12 hpi, with significant increases not occurring until 24 hpi and continuing through 48 hpi, reaching approximately 8.9 × 10^7^ TCID_50_/mL. Consistent with these kinetic differences, titers from mammalian-propagated infections were significantly higher than mosquito-propagated infections at both 12 and 24 hpi; however, this difference was no longer significant by 48 hpi, indicating that both conditions ultimately support comparable levels of productive infection.

*IFNβ* transcript induction was similarly accelerated in mammalian-propagated infections (Figure 1B). Mammalian-propagated infections produced a significant increase in *IFNβ* transcripts at 24 hpi, reaching approximately 2800-fold over mock-infected controls, and this level was maintained through 48 hpi. In contrast, mosquito-propagated infections produced no significant *IFNβ* transcript induction until 48 hpi, at which point levels reached approximately 1000-fold over mock-infected controls. *IFNβ* transcript levels were significantly higher in mammalian-propagated infections than mosquito-propagated infections at both 24 and 48 hpi.

These findings demonstrate that viral stock origin significantly impacts early infection kinetics and the magnitude of *IFNβ* transcript induction, consistent with prior reports that the host cell used to propagate alphaviruses can influence downstream innate immune responses in target cells [31,32]. Mosquito-propagated stocks were selected for all subsequent experiments because CHIKV is naturally transmitted to humans via *Aedes* mosquito saliva and mosquito-propagated stocks therefore more accurately reflect the biological context of primary human skin infection.

#### 3.1.2. CHIKV Infection Kinetics Are Reproducible Across hDF Donors

**Figure 2:**
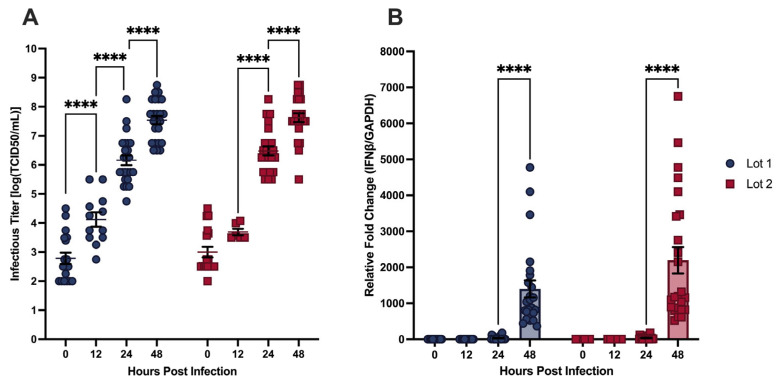
(**A**) Infectious CHIKV titers from the supernatants of infected hDFs from different donors at MOI 0.01 determined through TCID_50_. (**B**) Relative fold change of *IFNβ* transcript expression (*GAPDH* as endogenous control) of CHIKV-infected hDFs measured through RT-qPCR. Bars represent the mean, and error bars represent SEM. Data symbols represent individual samples. Individual samples were obtained from at least four separate experiments (*n* = 6 to 24). Statistical analysis using 2-way ANOVA with Šidàk’s multiple comparison test. **** *p* < 0.0001.

To assess whether findings from a single hDF donor could be generalized or responded differentially, as previously described [21], we compared two commercially available primary hDF lots derived from independent donors—referred to here as Lot 1 (CCD-1135Sk) and Lot 2 (CCD-1123Sk)—infected with mosquito-propagated CHIKV at MOI 0.01 over 48 hpi (Figure 2).

Infectious titer accumulation followed a broadly similar trajectory in both lots, with no significant differences observed between lots at any tested timepoint (Figure 2A). Within each lot, the titer increased significantly at each successive timepoint in Lot 1, from approximately 3.7 × 10^3^ TCID_50_/mL at 0 hpi to approximately 9.8 × 10^7^ TCID_50_/mL by 48 hpi. Lot 2 exhibited a similar overall trajectory and reached comparable titers by 48 hpi; however, unlike Lot 1, no significant titer increase was observed at 12 hpi, with significant accumulation not occurring until 24 hpi. Despite this difference in early accumulation kinetics, titers between the two lots were not significantly different at any timepoint.

*IFNβ* transcript expression was also comparable between lots, with both exhibiting near-zero levels through 12 hpi, modestly increasing at 24 hpi, and significantly increasing by 48 hpi (Figure 2B). Lot 1 reached approximately 1400-fold over mock-infected controls at 48 hpi, while Lot 2 reached approximately 2200-fold. Although Lot 2 trended toward higher *IFNβ* transcript levels than Lot 1 at 48 hpi, these values did not display statistically significant differences.

The absence of significant between-lot differences in either infectious titer or IFNβ transcript expression across the infection time course supports pooling data from both donor lots for subsequent analyses. All experiments described in Section 3.2 through Section 3.4 notably incorporate pooled data from Lot 1 and Lot 2 to maximize statistical power; therefore, donor-specific factors, including age, sex, health status, and ethnicity, may contribute to the modest difference in early titer accumulation kinetics observed between lots, but this difference did not affect overall infection outcomes or innate immune induction.

#### 3.1.3. MOI Determines the Timing of Viral Replication and Innate Immune Induction in hDFs

**Figure 3:**
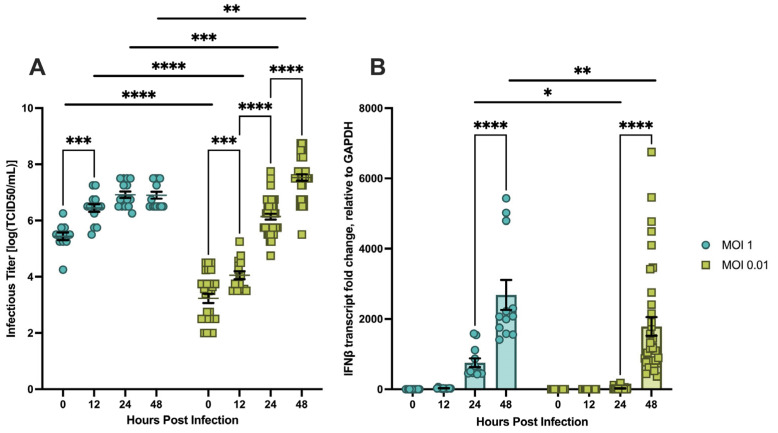
(**A**) Infectious CHIKV titers from the supernatants of infected hDFs at different MOIs determined through TCID_50_. (**B**) Relative fold change of *IFNβ* transcript expression (*GAPDH* as endogenous control) of CHIKV-infected hDFs measured through RT-qPCR. Bars represent the mean, and error bars represent SEM. Data symbols represent individual samples. Individual samples were obtained from at least four separate experiments (*n* = 12 to 36). Statistical analysis using 2-way ANOVA with Šidàk’s multiple comparison test. * *p* < 0.05 ** *p* < 0.01 *** *p* < 0.001 **** *p* < 0.0001.

To evaluate the impact of starting viral concentration on infection kinetics and innate immune induction, pooled hDFs from both donor lots were infected with mosquito-propagated CHIKV at MOI 1 or MOI 0.01 and monitored over 48 hpi (Figure 3).

Infectious titer differed significantly between the two MOIs at all four tested timepoints (Figure 3A). At MOI 1, titers began at approximately 4.2 × 10^5^ TCID_50_/mL at 0 hpi (reflective of the higher inoculum) and increased significantly to approximately 1.4 × 10^7^ by 12 hpi, with no significant changes observed at 24 or 48 hpi, indicating that highest measured titers were statistically similar to those reached at 12 hpi under these conditions. In contrast, MOI 0.01 infections began at approximately 7.1 × 10^3^ TCID_50_/mL and exhibited stepwise significant increases at each successive timepoint, reaching approximately 1 × 10^8^ TCID_50_/mL by 48 hpi. Notably, while MOI 1 produced higher titers at early timepoints, MOI 0.01 ultimately reached comparable or slightly higher titer levels by 48 hpi, suggesting greater cumulative viral production at the lower starting concentration over the duration of the time course. Consistent with greater early viral replication, MOI 1 produced significantly lower cell viability at 48 hpi compared to MOI 0.01 (Appendix A.2).

The timing of *IFNβ* transcript induction differed markedly between the two conditions (Figure 3B). At MOI 1, *IFNβ* transcript induction was observed as early as 24 hpi, with significant increases between 24 and 48 hpi, reaching approximately 2700-fold change over mock-infected controls. In contrast, MOI 0.01 infections produced no detectable *IFNβ* transcript induction at 12 or 24 hpi, with significant induction not observed until 48 hpi, at which point transcript levels reached approximately 1800-fold change. At 24 and 48 hpi, *IFNβ* transcript levels were significantly higher in MOI 1 infections than MOI 0.01 infections, demonstrating a dose-dependent delay in innate immune induction. Notably, all cells under both MOI conditions exhibited complete monolayer disruption by 72 hpi regardless of starting viral concentration (data not shown).

Although MOI 0.01 more closely approximates the inoculum deposited during natural mosquito feeding, MOI 1 was selected for subsequent mechanistic experiments for two reasons. First, MOI 1 produced detectable *IFNβ* transcript induction at earlier timepoints, providing greater temporal resolution of the innate immune response—a critical consideration for the mechanistic experiments described in Section 3.3 and Section 3.4. Second, MOI 1 is consistent with the multiplicity employed in other CHIKV in vitro studies [19,33,34,35], facilitating direct comparison of our findings with the existing literature.

### 3.2. CHIKV-Infected hDFs Mount a Robust but Translationally Insufficient IFNβ Response

**Figure 4:**
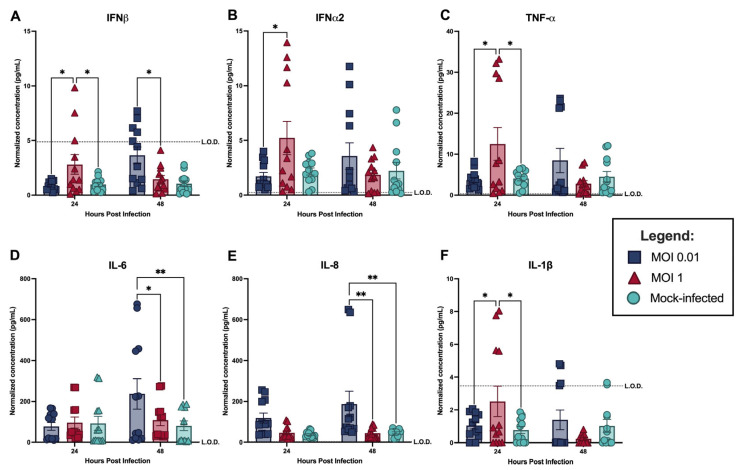
Cytokine profiles of CHIKV-infected hDF supernatants at different MOIs: (**A**) interferon-beta (IFNβ), (**B**) interferon-alpha 2 (IFNα2), (**C**) tumor necrosis factor-alpha (TNFα), (**D**) interleukin-6 (IL-6), (**E**) interleukin-8 (IL-8), (**F**) interleukin-1-beta (IL-1β). Data points represent the average concentrations of individual samples obtained from two separate experiments (*n* = 12). Bars represent the mean and error bars represent standard deviation (SD). Statistical analysis using 2-way ANOVA with Šidàk’s multiple comparison test. * *p* < 0.05 ** *p* < 0.01.

The standardization experiments described above reveal a striking inconsistency in the innate immune response of hDFs to CHIKV infection. At MOI 1, *IFNβ* transcript levels increased significantly as early as 24 hpi, reaching approximately 800-fold over mock-infected controls and continued to increase to approximately 1750-fold by 48 hpi (Figure 3B). However, average IFNβ protein concentrations in supernatants from MOI 1-infected hDFs did not exceed the limit of detection (LOD) of 4.5 pg/mL. Significant elevation over mock-infected controls was only observed at 24 hpi (Figure 4A). The protein levels achieved are demonstrably insufficient for antiviral protection—as shown in Section 3.4, a minimum of 20 pg/mL of exogenous IFNβ is required to rescue hDFs from lethal infection, a threshold that endogenous IFNβ production never approaches under any tested infection condition.

Beyond IFNβ, CHIKV infection of hDFs induced a broader pro-inflammatory cytokine response. IFNα2 was significantly elevated in MOI 1-infected supernatants at 24 hpi relative to mock-infected controls, though concentrations approached the LOD by 48 hpi (Figure 4B). TNFα was significantly elevated in MOI 1-infected supernatants at 24 hpi relative to both mock-infected and MOI 0.01 conditions, and this elevation was significantly reduced by 48 hpi (Figure 4C). IL-1β was similarly elevated in MOI 1-infected supernatants at 24 hpi relative to both mock-infected and MOI 0.01 conditions, though this significance was not maintained at 48 hpi (Figure 4F). IL-8 was significantly elevated in MOI 0.01-infected supernatants at 48 hpi relative to both mock-infected and MOI 1 conditions (Figure 4E). One notable and unexpected finding was the significant elevation of IL-6 in MOI 0.01-infected supernatants at 48 hpi relative to both mock-infected and MOI 1 conditions (Figure 4D). Upon separating data by donor lot, this elevation was attributable primarily to Lot 2 samples, suggesting that donor-specific factors may contribute to baseline IL-6 secretion in this lot (further elaborated on in the discussion).

Taken together, these findings establish the central paradox motivating the mechanistic experiments that follow: CHIKV-infected hDFs produce a transcriptionally robust but translationally insufficient IFNβ response, accompanied by secretion of pro-inflammatory cytokines, yet this response fails to prevent progressive viral replication and universal cell death by 72 hpi.

### 3.3. TBK1/IKKε Inhibition Does Not Suppress IFNβ Transcript Expression

**Figure 5:**
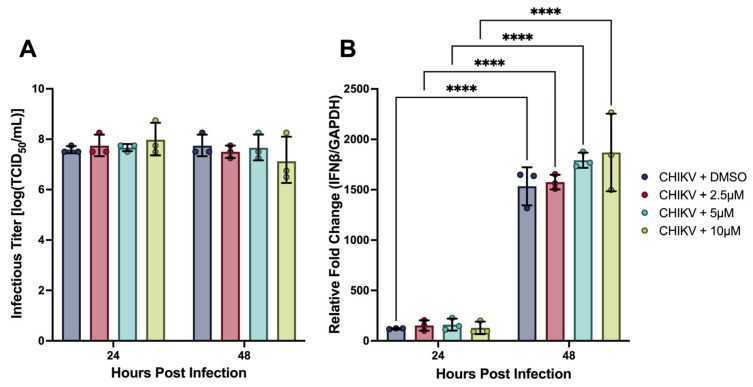
(**A**) Infectious CHIKV titers from the supernatants of infected hDFs co-inoculated with amlexanox, determined using TCID_50_. (**B**) Relative fold change of *IFNβ* transcript expression (*GAPDH* as endogenous control) of CHIKV-infected hDFs, measured using RT-qPCR. Bars represent the mean, and error bars represent SD. Data symbols represent individual biological replicates (*n* = 3). Statistical analysis using 2-way ANOVA with Šidàk’s multiple comparison test. **** *p* < 0.0001.

To interrogate whether canonical PRR signaling through TBK1/IKKε drives the robust IFNβ transcriptional response observed during CHIKV infection, hDFs were co-treated with amlexanox—a small molecule inhibitor of TBK1 and IKKε, which serve as convergent downstream effectors of both the RIG-I/MAVS and TLR3/TRIF signaling axes —alongside CHIKV at MOI 1 (Figure 5). Concentrations of 2.5, 5, and 10 µM were selected based on cytotoxicity screening in hDFs, which demonstrated that concentrations up to 100 µM did not significantly reduce cell viability in uninfected controls (Appendix A.3); however, concentrations above 10 µM were excluded from mechanistic experiments to remain within the range most consistently associated with selective TBK1/IKKε inhibition in cell-based systems. A DMSO vehicle control was included to account for solvent effects. Due to the preliminary nature of these experiments, each condition was assessed with three independent biological replicates.

Amlexanox treatment did not significantly affect infectious titer accumulation at either 24 or 48 hpi at any tested concentration relative to DMSO vehicle control, with all conditions producing titers of approximately 8 × 10^7^ TCID_50_/mL at both tested timepoints (Figure 5A). Cell viability did not differ significantly between any amlexanox-treated condition and CHIKV-alone controls at the concentrations tested, though a modest non-significant trend toward partial rescue was observed at lower concentrations (Appendix A.2).

Critically, *IFNβ* transcript expression did not differ significantly between any amlexanox concentration and DMSO vehicle control at either timepoint (Figure 5B). All four conditions exhibited comparable, near-zero transcript levels at 24 hpi, followed by significant and equivalent increases to approximately 1500–1800-fold over uninfected controls at 48 hpi. The consistency of this null result across three inhibitor concentrations, spanning a four-fold range from 2.5 to 10 µM, argues against a dose-dependent suppressive effect of TBK1/IKKε inhibition on *IFNβ* transcription.

These findings suggest that TBK1/IKKε-mediated signaling is not the major driver of IFNβ transcription in CHIKV-infected hDFs. Because TBK1 and IKKε are convergent downstream effectors shared by both the RIG-I/MAVS and TLR3/TRIF pathways (the two PRR axes most consistently implicated in IFNβ induction during CHIKV infection of nonhematopoietic cells) [15,17,26], this result indicates that neither pathway is the predominant source of the robust transcriptional response observed here. While these experiments were performed with limited replicates (*n* = 3) and pharmacological inhibition cannot fully exclude TBK1/IKKε-independent contributions of these receptors, the consistency of the null result across multiple inhibitor concentrations supports this interpretation. Genetic approaches, including MAVS or TRIF knockout, will be required to definitively resolve the contributions of individual PRR pathways.

### 3.4. Exogenous IFNβ Rescues hDFs from Lethal CHIKV Infection via Intact IFNAR Signaling

**Figure 6:**
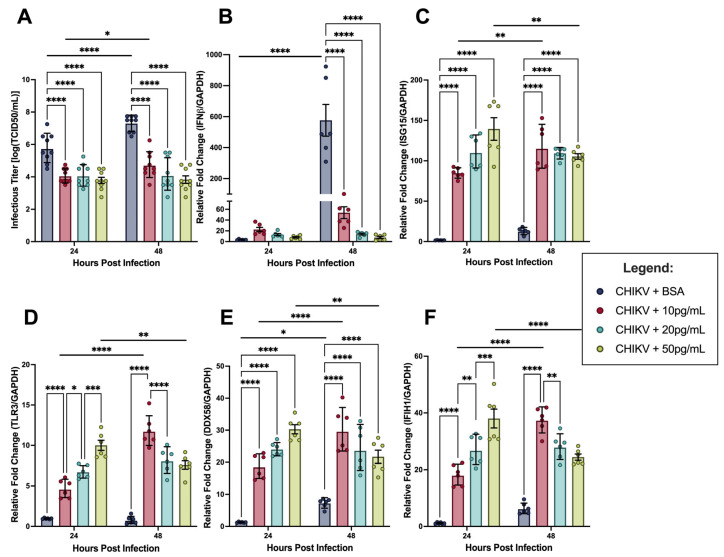
(**A**) Infectious CHIKV titers from the supernatants of infected hDFs co-inoculated with recombinant IFNβ determined through TCID_50_. (**B**–**F**) Relative fold change of (**B**) *IFNβ*, (**C**) *ISG15*, (**D**) *TLR3*, (**E**) *DDX58*, and (**F**) *IFIH1* transcript expression (*GAPDH* as endogenous control) of CHIKV-infected hDFs measured through RT-qPCR. Bars represent the mean, and error bars represent SEM. Data symbols represent individual samples from two separate experiments (*n* = 6). Statistical analysis using 2-way ANOVA with Šidàk’s multiple comparison test. * *p* < 0.05 ** *p* < 0.01 *** *p* < 0.001 **** *p* < 0.0001.

To determine whether hDFs retain functional IFNβ signaling capacity despite failing to mount a sufficient autocrine response, cells were co-inoculated with CHIKV at MOI 1 and recombinant human IFNβ at concentrations of 10, 20, or 50 pg/mL. A BSA vehicle control was included to account for the stabilizing agent present in the IFNβ preparation. Infectious titers, as well as IFNβ and ISG transcript expression, were measured at 24 and 48 hpi (Figure 6).

Consistent with intact IFNAR signaling, IFNβ prevented CHIKV titer accumulation in hDFs at 24 h and 48 h; at 24 hpi, titers in all IFNβ-treated wells were approximately 10^4^ TCID_50_/mL, and, by 48 hpi, infectious virus in the supernatants were suppressed to approximately 10^3^ TCID_50_/mL in cells treated with 50 pg/mL, 10^4^ TCID_50_/mL in cells treated with 20 pg/mL, and 10^5^ TCID_50_/mL in cells treated with 10 pg/mL by 48 hpi (Figure 6A). Conversely, infectious titers in the CHIKV-BSA wells averaged 10^6^ TCID_50_/mL at 24 hpi and 10^7^ TCID_50_/mL at 48 hpi. Additionally, no CPE accumulation was noted in any cells treated with IFNβ by 24 hpi. Interestingly, despite lacking widespread cell death as in the CHIKV-BSA wells, CHIKV-10 pg/mL IFNβ wells still accumulated titers from 24 to 48 h. These findings suggest that levels as low as 20 pg/mL of IFNβ prevent CHIKV infection of hDFs and levels as low as 10 pg/mL are sufficient to prevent widespread cell death.

Next, we measured the *IFNβ* transcript expression from the lysates of hDFs following IFNβ-CHIKV co-inoculation. While CHIKV-BSA-treated cells averaged 570-fold change expression compared to the negative control, CHIKV-IFNβ-treated cells averaged 53-fold change, 14-fold change, and 8-fold change with 10 pg/mL, 20 pg/mL, and 50 pg/mL of IFNβ, respectively, by 48 hpi (Figure 6B).

To further investigate the IFNβ signaling response in these cells, several ISGs were measured as well. For these analyses, *TLR3*, *DDX58* (or RIG-I), *IFIH1* (or MDA5), and *ISG15* were chosen (Figure 6C–F). While *TLR3* transcript expression averaged 3-fold change over the negative control in the CHIKV-BSA-treated cells at 24 hpi, approximately 15-fold change expression was observed in the CHIKV-IFNβ-treated wells regardless of treatment concentration. At 48 hpi, *TLR3* expression was unchanged in vehicle control infection. The 10 pg/mL and 20 pg/mL IFNβ-treated infections were significantly elevated over the CHIKV-BSA infections, reaching 25- and 22-fold change over uninfected controls (Figure 6D). In line with these findings, *DDX58* and *IFIH1* expression in CHIKV-BSA wells was significantly lower than all three IFNβ co-infected concentrations at 24 hpi; however, this significance is diminished by 48 hpi, though the trend is similar (Figure 6E,F). Finally, *ISG15* expression in IFNβ-treated infections was significantly higher than those of CHIKV-BSA infections. Interestingly, only CHIKV infections co-treated with 10 pg/mL of IFNβ expressed significant increases of *ISG15* transcripts between 24 h and 48 h (Figure 6C). This may reflect ongoing viral replication at the lowest IFNβ concentration, which continues to stimulate ISG expression between timepoints. Taken together with the infectious titer results, these data indicate that the hDFs used in these studies have intact IFNβ signaling responses.

## 4. Discussion

In this study, we systematically characterized CHIKV infection of primary hDFs and identified several findings that advance our understanding of early innate immune dynamics at the primary infection site. While it is established that CHIKV infects dermal fibroblasts and that type I interferon signaling broadly restricts alphavirus replication, the studies presented here make distinct contributions. First, we provide a systematic comparison of infection variables—viral stock origin, donor lot, and MOI—that are rarely evaluated in parallel and demonstrate that each significantly impacts infection kinetics and innate immune timing. Second, we identify a striking disconnect between IFNβ transcript induction, which reaches over 2800-fold above mock-infected controls, and IFNβ protein output, which peaks below 10 pg/mL—insufficient to establish an antiviral state. Third, we demonstrate that pharmacological inhibition of TBK1/IKKε, a convergent signaling node downstream of both RIG-I/MAVS and TLR3/TRIF, does not suppress this transcriptional response, suggesting that neither PRR pathway is its primary driver. Finally, we show that hDFs retain fully intact IFNAR signaling capacity, as exogenous IFNβ at physiologically low concentrations completely rescues cells from lethal infection and robustly induces ISG expression.

We confirmed that hDFs are, indeed, susceptible to CHIKV infection, as hDFs infected at MOIs of 1 and 0.01 accumulated significant titers over 48 h. Additionally, we describe how these cells express *IFNβ* transcripts that significantly increase throughout CHIKV infection relative to mock-infected cells (*GAPDH* as an endogenous control, reported to be most appropriate by Agrawal et al. [30]). This is in line with previous studies that utilized different fibroblast cell lines, primary and immortalized, as a model for CHIKV infection [17,19,20,23,24,36]. In addition, using two different lots of hDFs from two separate donors (obtained from ATCC), we found no significant differences in infectious titer or *IFNβ* transcript expression at any of the tested timepoints but did note a difference in the early growth kinetics between the two lots. Infections of “Lot 1” cells resulted in significant increases in infectious titer from baseline to 12 hpi, whereas significant increases in titer were not observed in “Lot 2” cells until 24 hpi. Several factors could contribute to this noted difference, including the donor’s age, sex, health status, and ethnicity. While we noted mild differences in the two hDF lots, others have found some hDF cells to be refractive to CHIKV infection [21,24]. This is contrary to our findings, as both cell lines exhibited no major differences. This could be explained by different dermal fibroblast subtypes. A recent study found that dermal fibroblasts exhibit six distinct subtypes in healthy skin [37], further complicating the interpretation of hDF infection by CHIKV.

We also found that the cell type used to generate viral stocks can drastically affect the results collected from CHIKV infection of hDFs. Early CHIKV studies produced viral stocks from mammalian cell lines, such as Vero E6 or BHK21, while more recent studies grow their viral stocks in mosquito cell lines like C6/36 and C7/10. The studies presented here provide evidence that the cell type used to produce viral stocks impacts the magnitude of infectious titer and *IFNβ* transcript expression. When comparing mammalian-propagated and mosquito-propagated viral stocks, infectious titer and *IFNβ* were significantly reduced in mosquito-propagated infections at 12 h and 24 hpi. While CHIKV titers reached similar endpoints at 48 hpi regardless of stock derivation, *IFNβ* transcript levels remained reduced in mosquito-propagated samples. This contrasts with Crawford et al., who found that mosquito-derived Sindbis virus (SINV), another mosquito-borne alphavirus, produced elevated levels of *IFNβ* transcript expression compared to mammalian-derived SINV. They also noticed no significant differences in infectious titer, whereas our studies found reduced titers from mosquito-derived CHIKV infection [31]. Notably, these studies utilized C7/10 cells—another *Aedes albopictus* larval derivation—for their mosquito-derived virus stocks, as well as BHK (or baby hamster kidney) cells for their mammalian-derived stocks. Ali et al. described genotypic changes to CHIKV following serial passage in different *Aedes*-derived cell lines [32]; however, they passaged the virus 30–100 times in these cells to study evolutionary mutations to adaptation over time, whereas the stocks generated in the studies presented here were derived from single passages in each cell line. Further studies are needed to establish the mechanisms behind the exhibited differences; however, our studies, taken with the previous studies described, underline the importance of considering which cell type is used to grow viral stocks. For example, when studying viral dissemination effects on downstream tissues, mammalian-derived stocks are likely more appropriate than mosquito-derived stocks; however, in studies like the ones presented here, mosquito-derived stocks more accurately represent natural CHIKV transmission to human skin cells.

As expected, we found vast differences in infection kinetics when comparing starting concentrations of CHIKV. Depending on the intention of the study, many MOIs have been used in CHIKV studies, from as low as MOI 0.001 to MOIs 50 and higher [17,23,38,39,40,41]. We measured differences in infectious titer at every timepoint tested, as well as differences in *IFNβ* transcript at 24 h and 48 hpi. Others have observed a similar disconnect in the transcriptional and translational responses we present here upon infecting human foreskin fibroblasts (HFFs) [25].

Interestingly, we noted that the cells from either MOI were completely detached from their wells by 72 hpi, suggesting that hDFs are highly permissive to CHIKV and unable to control infection alone. We hypothesize that hDFs in the skin could serve as infection signals to recruit immune cells to the initial site of infection. This is further confirmed by our studies, as CHIKV-infected hDFs secreted significant levels of TNFα, IL-1β, and IL-8. Future studies in our lab hope to explore which specific cell death pathways are activated, as well as how the presence of mosquito saliva may alter the observed phenotype.

We found that the hDF cell lines utilized in our studies are responsive to IFNβ treatment when inoculated simultaneously with CHIKV. While typical CHIKV infection of hDFs resulted in tremendous levels of infectious virus in the supernatants and widespread cell death by 48 h, simultaneous treatment with IFNβ kept CHIKV titers approximately three logs lower than those treated with BSA, as well as preventing cell death. This suggests that the cells established an antiviral state, protecting them from further infection and cell death. In addition, we found decreased levels of *IFNβ* transcript expression in cells treated with CHIKV and recombinant IFNβ, as well as significantly increased expression of ISGs, suggesting that exogenous IFNβ binds to IFNAR, signals through the JAK/STAT pathway, and increases the expression of *TLR3*, *DDX58*, *IFIH1*, and *ISG15* transcripts. Combined with the model evaluation results, these findings indicate that hDFs alone are unable to produce IFNβ cytokine levels at a magnitude or timing sufficient to prevent virus replication and the cells’ ultimate death, as IFNβ cytokine levels peaked at 10 pg/mL after 24 hpi when infected with CHIKV at an MOI of 1.

We propose that these results are consistent with IFNAR-induced secondary *IFNβ* transcript expression following minimal early innate signaling; however, due to host translational shutdown by CHIKV, the high transcript levels noted in these studies are not translated to high protein levels. This is in line with other studies that suggest CHIKV can shut down host transcriptional and translational machinery to favor viral replication over host defense. One study found that CHIKV nsP2 degrades Rpb1, a catalytic subunit of the RNA Polymerase II complex, which blocks host gene transcription [42]. Additionally, another group showed that CHIKV nsP2 inhibits STAT1 nuclear translocation following JAK/STAT signaling, preventing ISG expression (at least partially independent of host shut off) [43].

In contrast to findings in HFFs, where IFNβ induction was IPS-1/MAVS-dependent [25], pharmacological inhibition of TBK1/IKKε (the convergent downstream effector of both RIG-I/MAVS and TLR3/TRIF) did not suppress *IFNβ* transcription in hDFs, suggesting that the pathway architecture may differ by fibroblast subtype or cell derivation. The observation that TBK1/IKKε inhibition via amlexanox does not suppress *IFNβ* transcript induction raises an important mechanistic question about the source of the transcriptional response. TBK1 and IKKε are convergent downstream effectors shared by both the RIG-I/MAVS and TLR3/TRIF signaling axes, inducing IRF3 phosphorylation and dimerization, which leads to initiation of *IFNβ* transcription [26,44,45,46]—two PRR pathways most consistently implicated in CHIKV-driven IFNβ induction in fibroblasts [17,19,20,21,23,24,25]. The failure of amlexanox to suppress *IFNβ* transcription at any tested concentration, consistent across three inhibitor concentrations spanning a four-fold range, suggests that neither pathway serves as the primary driver of the transcriptional response observed here. While pharmacological inhibition cannot fully exclude TBK1/IKKε-independent contributions from these receptors, and while these experiments were performed with limited replicates (*n* = 3), the consistency of the null result supports this interpretation. Together with the IFNAR rescue data, these findings are consistent with a model in which minimal early innate sensing—potentially via low-level PRR activation—initiates limited IFNβ secretion that subsequently engages IFNAR to drive transcriptional amplification through JAK/STAT positive feedback. This amplified transcriptional response, while substantial at the transcript level, is ultimately rendered functionally insufficient by CHIKV-mediated translational suppression. Confirmation of this model will require direct testing, such as IFNAR blockade (via mechanisms like co-inoculation with anti-IFNAR1 neutralizing antibodies), to determine whether IFNAR signaling is necessary for the observed transcriptional amplification.

One abnormal finding in our study was the significant induction of IL-6 in mock-infected samples at 48 hpi compared to CHIKV-infected samples, as well as mock-infected samples from 24 hpi. While culture condition variability was considered, separation of the data by donor lot revealed that Lot 2 samples drove this elevation. Potential sources of this variation could be an inflammatory skin condition in this donor that does not produce significant morphological changes. Additionally, prior trauma to the area at the time of collection could have led to enrichment of activated fibroblasts. Finally, as mentioned previously, the cell lines could be derived from different hDF subtypes (i.e., reticular, papillary, or dermal–subcutaneous junction fibroblasts), which cannot be ruled out from the manufacturer-provided information.

Several limitations of this study warrant acknowledgment. First, as an in vitro system using a single cell type, this model does not recapitulate the complexity of human skin, which contains multiple permissive and non-permissive cell types whose interactions likely shape the early innate immune response. Second, the amlexanox experiments were performed with limited replicates (*n* = 3), and while the null result was consistent across three inhibitor concentrations, the range tested (2.5–10 µM) represents the lower boundary of concentrations used in some cell-based systems, despite falling above the reported cell-free IC_50_ of approximately 1–2 µM for TBK1. Confirmation with additional replicates, higher inhibitor concentrations, and genetic approaches (such as RIG-I, MAVS, or TRIF knockout systems) will be necessary to fully resolve the contributions of individual PRR pathways. Additionally, these studies lack a positive control to confirm pharmacological inhibition efficacy under experimental conditions (such as demonstrating amlexanox-mediated suppression of *IFNβ* induction following poly(I:C) stimulation), which would strengthen the interpretation that TBK1/IKKε inhibition was functionally achieved. Third, and critically, the IFNAR amplification model proposed here has not been directly tested; IFNAR blockade during active CHIKV infection is required to determine whether IFNAR signaling is necessary for the observed transcriptional response. Fourth, the absence of mosquito salivary components represents a deliberate scope limitation rather than an oversight—the infection system characterized here is intended to provide a standardized baseline against which salivary modulation can be evaluated in future studies. Finally, use of phenol-red-containing media throughout introduces a potential weak estrogenic stimulus via estrogen receptor activation in hDFs; while CHIKV-induced cell death suggests that this did not substantively alter our primary findings, phenol-red-free media should be considered for future experiments where fibroblast survival and proliferation are primary endpoints.

## 5. Conclusions

The studies presented here provide a systematic characterization of CHIKV infection in primary hDFs and reveal a critical disconnect between innate immune transcription and translation that may help explain the permissiveness of human skin fibroblasts during natural CHIKV infection. Using standardized infection conditions—including single-passage mosquito-propagated viral stocks, pooled primary hDF donor lots, and MOI 1—we demonstrate that hDFs support high-titered CHIKV replication accompanied by robust *IFNβ* transcript induction reaching up to approximately 2800-fold over mock-infected controls, yet IFNβ protein output never exceeds the threshold required for antiviral protection. Pharmacological inhibition of TBK1/IKKε via amlexanox did not suppress this transcriptional response, suggesting that neither RIG-I/MAVS nor TLR3/TRIF signaling—the two PRR axes most consistently implicated in CHIKV-driven IFNβ induction—serves as the primary driver of the observed transcriptional activity. Together with the demonstration that hDFs retain fully intact IFNAR signaling capacity, these findings are consistent with a model in which minimal early innate sensing initiates limited IFNβ secretion, which is subsequently amplified through IFNAR-JAK/STAT positive feedback, while CHIKV-mediated translational suppression prevents this amplified transcriptional response from producing sufficient protein to establish an antiviral state.

These results have direct implications for understanding early CHIKV pathogenesis in human skin. The failure of hDFs to protect themselves from lethal infection appears to reflect a production defect rather than a signaling defect—these cells can respond robustly to exogenous IFNβ but cannot produce sufficient concentrations of their own. This suggests that in the natural infection context, protection of dermal fibroblasts may depend on paracrine IFNβ signaling from neighboring cell types, including tissue-resident immune cells or keratinocytes, rather than autocrine protection. Future studies incorporating IFNAR blockade during active infection will be necessary to directly test the proposed amplification model, and ex vivo skin explant or co-culture systems will be needed to evaluate how the broader skin microenvironment—including mosquito salivary components—modulates these dynamics. Together, the infection system characterized here provides a rigorous and reproducible foundation for these future investigations.

## Figures and Tables

**Figure A1:**
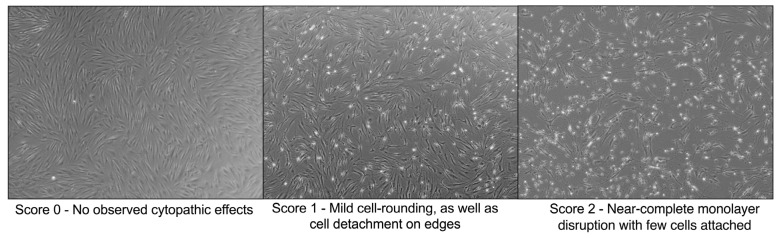
Representative images of typical CPE accumulation noted in CHIKV-infected hDFs. Score 0 represents no CPE, which displays uninfected controls (**left panel**). Score 1 represents the beginning of CPE accumulation, with mild cell rounding and detachment of cells, especially along cell edges (**middle panel**). Score 2 represents severe CPE accumulation noted by 48 hpi, with near complete monolayer disruption and few cells remaining attached to the culture surface (**right panel**).

**Figure A2:**
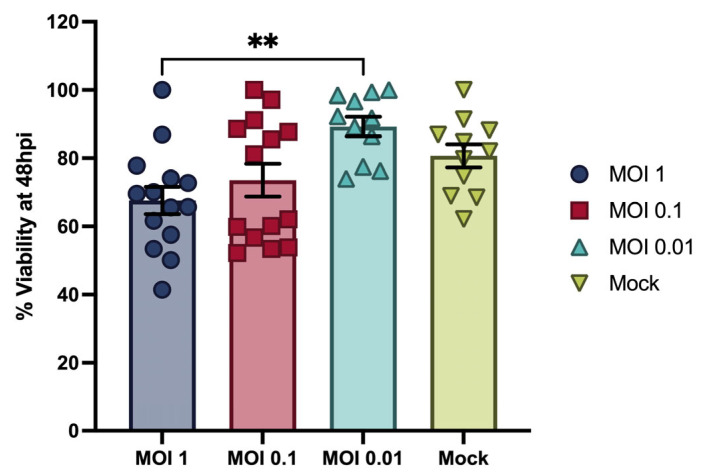
Cell viability of hDFs infected with CHIKV at indicated MOIs or mock-infected, assessed at 48 hpi. Bars represent the mean, and error bars represent SD. Data symbols represent individual samples. MOI 1 produced significantly lower viability than MOI 0.01 (** *p* = 0.0028). Statistical analysis using 1-way ANOVA with Šidàk’s multiple comparison test.

**Figure A3:**
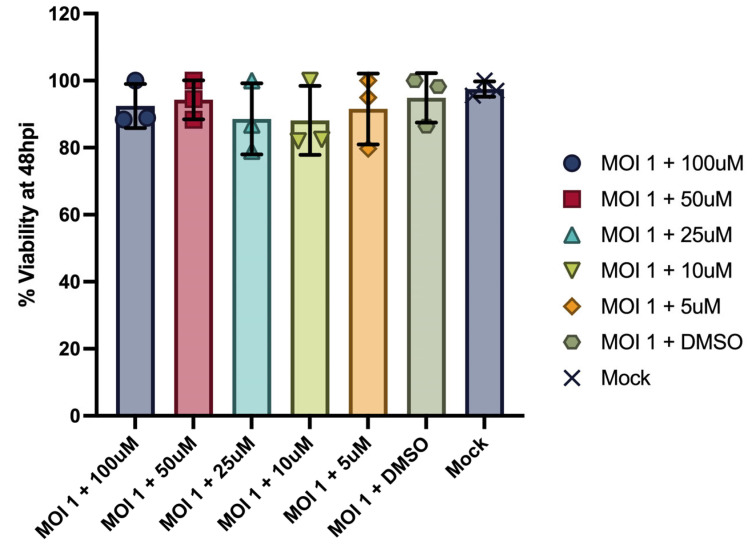
Cell viability of hDFs infected with CHIKV at MOI 1 in the presence of amlexanox at indicated concentrations or mock-infected controls, assessed at 48 hpi. Bars represent the mean, and error bars represent SD. Data symbols represent individual condition wells (*n* = 3). No significant differences were observed between any amlexanox-treated condition and CHIKV-DMSO controls, nor any infected conditions with mock-infected controls. Statistical analysis using 1-way ANOVA with Šidàk’s multiple comparison test.

## Data Availability

The raw data supporting the conclusions of this article will be made available by the authors upon request.

## Supplementary Materials

The following supporting information can be downloaded at https://www.mdpi.com/article/10.3390/v18050503/s1, File S1: Primer sequences and ATCC-provided hDF information. File S2: RT-qPCR gene expression template; File S3: TCID_50_ scoring template.

## Appendix A

### Appendix A.2. Cell Viability Data of hDFs Infected with CHIKV at Different MOIs

Viability was determined using CellTiter-Glo 2.0 (Promega, Madison, WI, USA; G9242) per the manufacturer’s instructions. Briefly, hDFs were seeded into black-walled 96-well plates for next-day use. Cells were inoculated with the indicated MOI in maintenance media and then incubated at 37 °C with 5% CO_2_ until 48 hpi. At 48 hpi, the plate was equilibrated at room temperature for 30 min, and then CellTiter-Glo 2.0 reagent was added in equal volumes to culture media. The plates were then placed into a Dynex Triad plate reader and orbitally shaken for 2 min to induce cell lysis. Plates were then incubated in the plate reader for 10 min to stabilize luminescent signal, and then luminescence was recorded. Viability was determined by normalizing relative light units (RLUs), where 100% viability was determined by the highest RLUs in the uninfected control wells and 0% based on cell-free luminescent wells.
