# Supplementary material for: CHIKV-Infected Human Dermal Fibroblasts Mount an IFNβ Transcriptional Response Independent of TBK1/IKKε Signaling That Fails to Prevent Lethal Infection
Source: Viruses. 2026 Apr 28;18(5):503. doi: 10.3390/v18050503 (PMC13211683; doi:10.3390/v18050503)
Supplement: Supplementary file 1 [file viruses-18-00503-s001.zip › SupplementaryFile S1.pdf]

Supplementary Materials File S1:

PCR Primer and Probe Sequences:

Target	Primer/Probe	Sequence
**IFNβ**	Forward	GCCATCAGTCACTTAAACAGC
	Reverse	GAAACTGAAGATCTCCTAGCCT
	Probe	/5'6-FAM/ TGAAGCAAT /ZEN/ TGTCCAGTCCCAGAGG /3'IABkFQ/
**IDT IFNβ Pre-designed Assay Name:** Hs.PT.58.39481063.g		
**GAPDH**	Forward	TGTAGTTGAGGTCAATGAAGGG
	Reverse	ACATCGCTCAGACACCATG
	Probe	/56-FAM/ AAGGTCGGA /ZEN/ GTCAACGGATTTGGTC /3IABkFQ/
**IDT GAPDH Pre-designed Assay Name:** Hs.PT.39a.22214836		
**CHIKV**	Forward	TCACTCCCTGTTGGACTTGATAGA
	Reverse	TTGACGAACAGAGTTAGGAACATACC
	Probe	AGGTACGCGCTTCAAGTTCGGCG

ATCC-provided Details on hDF donors:

<u>hDF1 (CCD-1135Sk)</u>

RRID:CVCL_2370
Sex: Male
Ethnicity: White
Age: 34
Comments: The cell line was derived from normal skin. Cells senesce after approx. 46 population doublings.

<u>hDF2 (CCD-1123Sk)</u>

RRID:CVCL_2361
Sex: Female
Ethnicity: not provided
Age: 40
Comments: The cell line was derived from normal skin. Cells senesce after approx. 54 population doublings.

Expanded Methods Information

Cells

Master stocks of hDFs were grown as described by the manufacturer in Iscove's Modified Dulbecco's Medium (IMDM) (ATCC, 30-2005) with 10% heat-inactivated fetal bovine serum (FBS) (BioWest, S160) and 1% penicillin/streptomycin (Gibco, 15140-122) at 37°C with 5% CO2. Stocks of cells were first slowly frozen down in a Mr. Frosty Box (ThermoScientific, 5100-1000) at -80°C overnight, then moved to the vapor phase of a liquid nitrogen dewar. FBS was heat-inactivated by incubating at 56.4°C for 30min with regular agitation. Working stocks were generated by thawing a single vial of the master stocks, growing

them in IMDM-based complete media, then after two passages, IMDM-based media was swapped for DMEM-based complete media. To detach hDF cells and Vero E6 cells from tissue culture flasks, 1mL per 25cm2 of TrypLE Express (Gibco, 12604-021) was added on adherent cells and incubated at 37°C with 5% CO2 for 10min or until most cells detached. To detach C6/36's, cells were gently scraped with cell-scraper (FisherScientific, 08-100-241), then collected in L15-based media. Cultures for all cell types were stopped prior to passage 20 and restarted with working stocks. Tissue cultures flasks were reutilized for maintenance of cultures between plate seedings; however, this was limited to three passages before acquiring a new culture flask. Cells, except hDFs, were centrifuged following trypsinization, then resuspended in fresh media prior to seeding. Cells counts were achieved by adding appropriate dilution of cell suspension (1:2 for hDF cells; 1:10 for C6/36 cells; 1:20 for Vero E6 cells) to trypan blue, then adding the combined trypan blue-cell suspension to both sides of a reusable hemacytometer.

hDF infection with CHIKV

Upon preparing viral inoculums with calculated dilutions, each well was inoculated with 0.3mL with appropriate viral dilutions. Plates were returned to 37°C with 5% CO2 for 1h with periodic agitation of plates (~10-15 min). After the 1h adsorption, maintenance media was added without removing the inoculum.

RT-qPCR

CHIKV genomic equivalents from supernatants was determined by preparing a 10-fold serial dilution of RNA extracted from CHIKV viral stocks, spanning from 10^{11} copies/mL to 10^{5} copies/mL. Genomic equivalents were determined by extrapolating concentration from this standard curve using QuantStudio Design and Analysis v2.8.0.
